# Rheumatoid arthritis increases the risk of pleural empyema

**DOI:** 10.1515/med-2020-0225

**Published:** 2020-10-08

**Authors:** Kuang-Ming Liao, Cheng-Li Lin, Te-Chun Shen

**Affiliations:** Department of Internal Medicine, Chi Mei Medical Center, Chiali, Tainan, Taiwan; Management Office for Health Data, China Medical University Hospital , Taichung, Taiwan; Division of Pulmonary and Critical Care Medicine, Department of Internal Medicine, China Medical University Hospital, No. 2, Yude Road, Taichung, 404, Taiwan; School of Medicine, China Medical University, Taichung, Taiwan

**Keywords:** pleural empyema, rheumatoid arthritis, retrospective cohort study

## Abstract

**Background:**

Rheumatoid arthritis (RA) can lead to various pulmonary manifestations. Evidence shows the possible association between RA and pleural empyema.

**Methods:**

We conducted a retrospective cohort study to investigate the risk of pleural empyema in patients with RA. The RA group (*n* = 29,061) included newly diagnosed adult patients between 2000 and 2012. The comparison group (*n* = 1,16,244) included individuals without RA at a 1:4 ratio of frequency matched by age, gender, and diagnosis year. The occurrence of pleural empyema was monitored until the end of 2013.

**Results:**

Patients with RA had a higher risk of developing pleural empyema than those without RA (23.6 vs 1.82 per 10,000 person-years, adjusted hazard ratio = 11.0, 95% confidence interval [CI] = 8.90–13.5). Furthermore, intensive care unit admission rates of pleural empyema were 37.7% in the RA group and 37.2% in the comparison group (adjusted odds ratio [OR] = 1.02, 95% CI = 0.66−1.57). The 30-day mortality rates of pleural empyema were 11.2% in the RA group and 10.9% in the comparison group (adjusted OR = 1.01, 95% CI = 0.51−1.88).

**Conclusion:**

Patients with RA are at a greater risk of developing pleural empyema than those without RA.

## Abbreviations

CCI, Charlson comorbidity index
CI, confidence interval
CKD, chronic kidney disease
CLD, chronic liver disease and cirrhosis
COPD, chronic obstructive pulmonary disease
HR, hazard ratio
ICD-9-CM, international classification of diseases, 9th revision, clinical modification
ICU, intensive care unit
LHID2000, longitudinal health insurance database 2000
NHI, national health insurance
NHIRD, national health insurance research database
OR, odds ratio
PY, person-year
RA, rheumatoid arthritis
SD, standard deviation

## 1 Introduction

Rheumatoid arthritis (RA) is a systemic disease involving inflammatory reaction of the immune system against body tissues, frequently leading to joint destruction and physical disability [1]. Extra-articular manifestations in RA are common and can occur at any age, primarily involving the skin, eyes, brain, heart, lungs, and kidneys [2]. Pleuropulmonary complications of RA include the following: (a) noninfective complications such as parenchymal involvement, airway involvement, vascular involvement, and pleural involvement; (b) infective complications such as pneumonia, abscess, bronchitis, and bronchiectasis; and (c) drug-related complications such as methotrexate-, gold-, sulfasalazine-, and azathioprine-induced adverse effects [3,4]. Pleural involvement is common, but usually without symptoms; autopsy studies reported pleural effusion in 50% of cases, with only 10% clinically detected [2,5]. RA-related pleural effusion usually exudates with mixed cell counts, low pH levels, low glucose levels, high lactate dehydrogenase levels, and high protein levels [4].

Pleural empyema refers to frank pus in the pleural space [6]. The most common etiology is secondary to pneumonia [7]. As expected, mortality is much higher in pneumonia patients with pleural empyema than in those without pleural empyema [8,9]. The clinical significance of pleural empyema and the importance of its drainage have been identified for many centuries [10]. Delay diagnosis and inadequate drainage are associated with substantially higher mortality [11]. Alcoholism, drug abuse, diabetes mellitus, immunocompromised status, cancer, pulmonary disease, and preexisting pleural effusion are common risk factors for the development of pleural empyema [12,13].

To date, there have been several case reports and case series [14,15,16,17,18] that focus on the development of pleural empyema in patients with RA. The detailed mechanisms explaining the association remained unclear; however, some hypotheses could be suggested. First, RA itself and therapeutic agents could influence the immune function that promotes the susceptibility to various infections, including pneumonia and other respiratory infections [19]. Second, chronic rheumatoid pleural effusion could provide a potential environment for microbial translocation and growth [4,18]. Repeat diagnostic and therapeutic thoracentesis may also predispose patients to infection by providing a portal of entry to the pleural cavity. Third, colonization of necrotizing subpleural rheumatoid nodules may contribute to the development of pleural empyema [4,20]. Finally, RA-related pulmonary disorders such as parenchymal nodules, interstitial lung disease, airway disease, and pulmonary vasculitis may play a role in respiratory infections and pleural empyema [3,4].

Since pleural empyema is an essential infection of the respiratory system and requires timely treatments, it is important to investigate the risk of pleural empyema in patients with RA. The aim of this study is to examine whether patients with RA are at an increased risk of incident pleural empyema. In addition, we further evaluated the prognosis of pleural empyema between individuals with and without RA.

## 2 Materials and methods

### 2.1 Data source

The National Health Insurance (NHI) program, established in 1995, covered more than 99.9% of Taiwan’s residents to date. The National Health Research Institutes manages and updates the NHI Research Database (NHIRD). We used the Longitudinal Health Insurance Database 2000 (LHID2000, a subset of NHIRD) for this study, which included medical claims of 1 million people randomly selected in 2000. All information regarding demographic status, diagnostic codes, medication claims, and procedure claims was available in LHID2000. This study was approved by the Research Ethics Committee of the China Medical University and Hospital (CMUH-104-REC2-115).

### 2.2 Study population

The RA group included adult patients with newly diagnosed RA (International Classification of Diseases, 9th Revision, Clinical Modification [ICD-9-CM] 714.0) between 2000 and 2012, and the date of diagnosis was defined as the index date. We excluded those who had been diagnosed with pleural empyema before the index date and those with incomplete age or gender information. The comparison group included individuals without RA at a 1:4 ratio of frequency matched by age, gender, and index year. The exclusion criteria for the comparison group were the same as those for the RA group. All participants were monitored until the development of pleural empyema, withdrawal from NHI, death, or the end of 2013, whichever came first.

### 2.3 Outcome and comorbidities

The primary interest of this study was the occurrence of pleural empyema (ICD-9-CM code 510) [21,22]. Only empyema cases that received adequate and sufficient antimicrobial treatment could be confirmed as an empyema event in this study. Furthermore, we evaluated the intensive care unit (ICU) admission and 30-day mortality rates among pleural empyema events in the RA and comparison groups. We collected several comorbidities related to pleural empyema as potential confounders, which included diabetes mellitus (ICD-9-CM code 250), asthma/chronic obstructive pulmonary disease (COPD; ICD-9-CM codes 493 and 496), chronic liver disease and cirrhosis (CLD; ICD-9-CM code 571), chronic kidney disease (CKD; ICD-9-CM code 585), stroke (ICD-9-CM codes 430–438), cancer (ICD-9-CM codes 140–209), malnutrition (ICD-9-CM codes 260–269), alcohol-related diseases (ICD-9 codes 291, 303, 305.0, 790.3, and V11.3), drug dependence or abuses (ICD-9-CM codes 304, 305.1–305.9), and disorders involving the immune mechanism (ICD-9-CM code 279). In addition, we used the Charlson Comorbidity Index (CCI) score to assess the comorbidity level.

### 2.4 Statistical analysis

We compared the distribution of baseline characteristics between the RA and comparison groups by using the chi-square test for categorical variables and the *t*-test for continuous variables. The Kaplan–Meier curve showed the cumulative incidence of pleural empyema in both groups, and the difference was tested by the log-rank test. Hazard ratios (HRs) and 95% confidence intervals (CIs) were estimated using Cox proportional hazard models. The multivariate Cox proportional hazard model was applied to estimate the adjusted HRs (aHRs) after controlling for age, gender, comorbidity, and CCI score, which were significant in the univariate model. For the prognosis analysis, crude and adjusted odds ratios (ORs) were estimated using logistic regression models. The data were analyzed using the SAS statistical software (Version 9.4 for Windows; SAS Institute, Inc., Cary, NC, USA). A statically significant difference was set as *p*-value less than 0.05.

**Ethics approval and consent to participate:** This study was granted the approval by the Research Ethics Committee of the China Medical University and Hospital (CMUH-104-REC2-115).

**Consent for publication:** Not applicable.

## 3 Results

We recruited the RA and comparison groups consisting of 29,061 and 1,16,244 individuals, respectively. Table 1 presents the distribution of RA and non-RA and the percentage of associated comorbidities in both groups with details and significance. The distributions of the age group and gender did not differ between the RA and comparison groups. The mean age ± standard division of the RA group was 54.1 ± 14.0 years. In both groups, 77.6% of the individuals were women. Compared with individuals without RA, patients with RA had significantly higher prevalence for CLD (23.6% vs 17.7%, *p* < 0.001), asthma/COPD (12.5% vs 8.48%, *p* < 0.001), CKD (10.5% vs 1.43%, *p* < 0.001), disorders involving the immune mechanisms (6.85% vs 0.29%, *p* < 0.001), stroke (2.98% vs 3.44%, *p* < 0.001), alcohol-related diseases (0.83% vs 0.44%, *p* < 0.001), and drug dependence or abuses (0.33% vs 0.09%, *p* < 0.001). Compared with individuals without RA, patients with RA had a trend to have a higher CCI score (*p* < 0.001).

**Table 1:** Baseline characteristics of patients with RA and individuals without RA

	Non-RA		RA		
	*N* = 116,244		*N* = 29,061		
	*n*	%	*n*	%	*p*-value
**Age (years)**					0.99
20–49	45,160	38.9	11,290	38.9	
50–64	43,916	37.8	10,979	37.8	
≥65	27,168	23.4	6,792	23.4	
Mean ± SD	53.6	±14.3	54.1	±14.0	<0.001
**Gender**					0.99
Women	90,228	77.6	22,557	77.6	
Men	26,016	22.4	6,504	22.4	
**Comorbidity**					
Diabetes mellitus	10,930	9.40	2,668	9.18	0.25
Asthma/COPD	9,863	8.48	3,632	12.5	<0.001
CLD	20,576	17.7	6,857	23.6	<0.001
CKD	1,659	1.43	3,040	10.5	<0.001
Stroke	4,000	3.44	866	2.98	<0.001
Cancer	3,177	2.73	717	2.47	0.01
Malnutrition	666	0.57	182	0.63	0.29
Alcohol-related diseases	512	0.44	241	0.83	<0.001
Drug dependence or abuses	107	0.09	97	0.33	<0.001
Disorders of immune mechanism	332	0.29	1991	6.85	<0.001
**CCI score** **a**					<0.001
0	1,02,489	88.2	21,262	73.2	
1	8,290	7.13	5,114	17.6	
2	2,653	2.28	1,589	5.47	
3 or more	2,812	2.42	1,096	3.77	

CCI, Charlson Comorbidity Index; CKD, chronic kidney disease; CLD, chronic liver disease and cirrhosis; COPD, chronic obstructive pulmonary disease; RA, rheumatoid arthritis; SD, standard deviation.

^a^CCI score calculation did not include connective tissue disease.

The overall incidence density rates of pleural empyema were 23.6 and 1.82 per 10,000 person-years in the RA and comparison groups, respectively (Table 2). Compared with that in the comparison group, the corresponding aHR for pleural empyema was 11.0 (95% CI = 8.90–13.5) in the RA group after adjusting for age, gender, comorbidity, and CCI score. Compared with that in individuals aged 20–49 years, the risks of pleural empyema were 2.29- and 6.07-fold higher than in those aged 50–64 years (95% CI = 1.71–3.06) and ≥65 years (95% CI = 4.58–8.05), respectively. Furthermore, the risk of pleural empyema was higher in individuals with diabetes mellitus (aHR = 1.54, 95% CI = 1.23–1.91), asthma/COPD (aHR = 1.38, 95% CI = 1.12–1.69), cancer (aHR = 1.27, 95% CI = 1.05–1.55), and CKD (aHR = 1.26, 95% CI = 1.00–1.60) than in those without these comorbidities. Compared with that in individuals with a CCI score of 0, the risks of pleural empyema were 1.72-, 2.42-, and 3.47-fold higher than in those with CCI scores of 1 (95% CI = 1.38–2.14), 2 (95% CI = 1.81–3.22), and 3 or more (95% CI = 2.54–4.73).

**Table 2:** Potential risk factors for pleural empyema

	Event	PY	Rate^a^	Crude HR (95% CI)	Adjusted HR^b^ (95% CI)
**Age (years)**					
20–49	66	3,61,939	1.82	1.00	1.00
50–64	165	3,35,482	4.92	2.70 (2.03–3.59)***	2.29 (1.71–3.06)***
≥65	309	1,83,991	16.8	9.27 (7.11–12.1)***	6.07 (4.58–8.05)***
**Gender**					
Women	328	6,96,097	4.71	1.00	1.00
Men	212	1,85,316	11.4	2.43 (2.05–2.89)***	1.98 (1.66–2.36)***
**Comorbidity**					
Non-RA	129	7,07,470	1.82	1.00	1.00
RA	411	1,73,942	23.6	13.0 (10.6–15.8)***	11.0 (8.90–13.5)***
No-DM	431	8,09,977	5.32	1.00	1.00
DM	109	71,436	15.3	2.88 (2.33–3.55)***	1.54 (1.23–1.91)***
Non-asthma/COPD	409	8,09,138	5.05	1.00	1.00
Asthma/COPD	131	72,275	18.1	3.60 (2.96–4.38)***	1.38 (1.12–1.69)***
Non-CLD	391	7,20,038	5.43	1.00	1.00
CLD	149	1,61,375	9.23	1.70 (1.41–2.05)***	0.97 (0.80–1.18)
Non-CKD	445	8,56,162	5.20	1.00	1.00
CKD	95	25,251	37.6	7.25 (5.81–9.04)***	1.26 (1.00–1.60)*
Non-stroke	490	8,58,446	5.71	1.00	1.00
Stroke	50	22,967	21.8	3.83 (2.86–5.13)***	0.97 (0.70–1.34)
Non-cancer	401	7,08,892	5.66	1.00	1.00
Cancer	139	1,72,521	8.06	1.43 (1.18–1.73)***	1.27 (1.05–1.55)*
No-malnutrition	536	8,76,834	6.11	1.00	
Malnutrition	4	4,579	8.74	1.43 (0.54–3.83)	
Non-alcohol-related diseases	535	8,77,936	6.09	1.00	
Alcohol-related diseases	5	3,477	14.4	2.37 (0.98–5.71)	
Non-drug dependence or abuses	537	8,80,560	6.10	1.00	1.00
Drug dependence or abuses	3	853	35.2	5.79 (1.86–18.0)**	2.58 (0.82–8.06)
Non-immunodeficiency	513	8,68,530	5.91	1.00	1.00
Disorders of immune mechanism	27	12,883	21.0	3.55 (2.41–5.23)***	1.19 (0.81–1.77)
**CCI score** c					
0	266	76,642	3.47	1.00	1.00
1	132	77,437	17.1	4.92 (3.99, 6.06)***	1.72 (1.38, 2.14)***
2	66	21,568	30.6	8.88 (6.78, 11.6)***	2.42 (1.81, 3.22)***
3 or more	76	16,766	45.3	13.2 (10.3, 17.1)***	3.47 (2.54, 4.73)***

CCI, Charlson Comorbidity Index; CI, confidence interval; CKD, chronic kidney disease; CLD, chronic liver disease and cirrhosis; COPD, chronic obstructive pulmonary disease; HR, hazard ratio; PY, person-years; RA, rheumatoid arthritis.

^a^Incidence rate per 10,000 person-years.

^b^CCI score calculation did not include connective tissue disease.

^c^Multivariable analysis including age, sex, and comorbidities of diabetes mellitus, asthma/COPD, CLD, CKD, stroke, cancer, drug dependence or abuses, disorders of immune mechanism, and CCI score; **p < 0.01, ***p < 0.001.

The association between RA and pleural empyema stratified by age, gender, and comorbidity presence is presented in Table 3. The aHRs for pleural empyema in the RA group were 8.30 (95% CI = 4.68–14.7), 8.38 (95% CI = 5.86–12.0), and 13.9 (95% CI = 10.5–18.5) in individuals aged 20–49, 50–64, and ≥65 years compared with that in the comparison group, respectively. The aHRs for pleural empyema were 14.6 (95% CI = 10.9–19.2) and 8.06 (95% CI = 5.93–11.0) in women and men in the RA group compared with that in the comparison group, respectively. In addition, the aHRs for pleural empyema were 10.1 (95% CI = 6.99–14.7) and 13.3 (95% CI = 10.5–16.9) in individuals without and with any comorbidity in the RA group compared with that in the comparison group, respectively.

**Table 3:** Incidences and hazard ratios of empyema for patients with RA compared to those without RA

	Non-RA		RA		Crude HR (95% CI)	Adjusted HR^a^ (95% CI)
	Event	Rate^b^	Event	Rate^b^		
**Age (years)**						
20–49	18	0.62	48	6.57	10.6 (6.14–18.1)***	8.30 (4.68–14.7)***
50–64	45	1.67	120	18.1	10.8 (7.70–15.3)***	8.38 (5.86–12.0)***
≥65	66	4.41	243	70.5	16.1 (12.2–21.1)***	13.9 (10.5–18.5)***
**Gender**						
Women	64	1.15	264	19.1	16.7 (12.7–22.0)***	14.5 (10.9–19.2)***
Men	65	4.35	147	40.8	9.36 (6.99–12.5)***	8.06 (5.93–11.0)***
**Comorbidity**						
Without comorbidity	45	1.11	72	10.6	9.55 (6.58–13.9)***	10.1 (6.99–14.7)***
With any comorbidity	84	2.78	339	32.0	11.5 (9.09–14.7)***	13.3 (10.5–16.9)***

CI, confidence interval; HR, hazard ratio; PY, person-years; RA, rheumatoid arthritis; ***p < 0.001.

^a^Multivariable analysis including age, sex, and comorbidities of diabetes mellitus, asthma/COPD, CLD, CKD, stroke, cancer, drug dependence or abuses, disorders of immune mechanism, and CCI score.

^b^Incidence rate per 10,000 person-years

**Figure 1:**
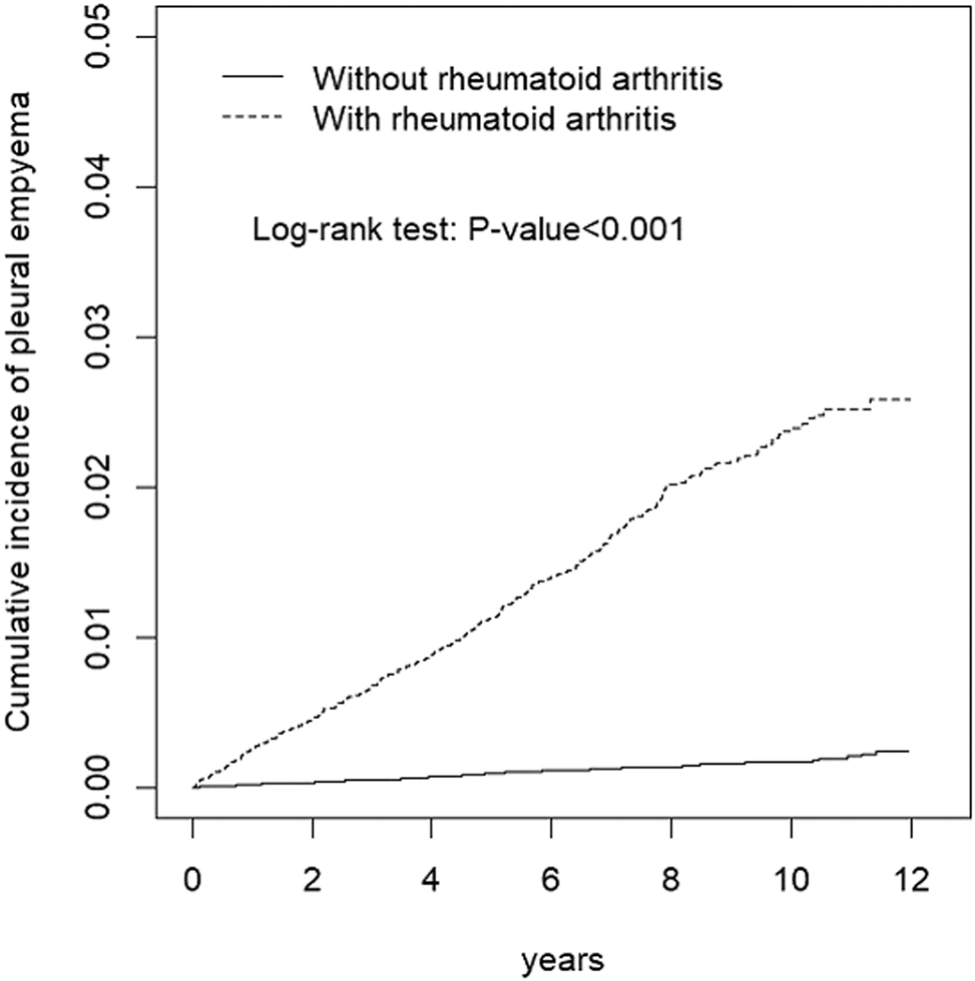
Cumulative incidence of empyema between individuals with and without rheumatoid arthritis.

In our analysis of prognosis (Table 4), we found that the ICU admission rate for pleural empyema was higher, but not significantly, in the RA group than in the comparison group (37.7% vs 37.2%, adjusted OR = 1.02, 95% CI = 0.66–1.57). In addition, the 30-day mortality rate for pleural empyema was higher, but not significantly, in the RA group than in the comparison group (11.2% vs 10.9%, adjusted OR = 1.01, 95% CI = 0.51–1.88). Cumulative incidences of pleural empyema in individuals with and without RA are illustrated in Figure 1. Patients with RA had a significantly higher cumulative incidence of pleural empyema than those without RA (log-rank test, *p* < 0.0001).

**Table 4:** ICU admission rate and 30-day mortality rate of empyema for patients with RA compared to those without RA

	Non-RA	RA
**ICU admission**		
ICU admission/empyema events	48/129	155/411
ICU admission rate	37.2%	37.7%
Crude OR (95% CI)	1 (reference)	1.02 (0.68–1.54)
Adjusted OR^a^ (95% CI)	1 (reference)	1.02 (0.66–1.57)
**Mortality**		
Mortality/empyema events	14/129	46/411
Mortality rate	10.9%	11.2%
Crude OR (95% CI)	1 (reference)	1.03 (0.55–1.95)
Adjusted OR^a^ (95% CI)	1 (reference)	1.01 (0.51–1.88)

CI, confidence interval; ICU, intensive care unit; OR, odds ratio; RA, rheumatoid arthritis.

^a^Multivariable analysis including age, sex, and comorbidities of diabetes mellitus, asthma/COPD, CLD, CKD, stroke, cancer, drug dependence or abuses, disorders of immune mechanism, and CCI score.

## 4 Discussion

This retrospective population-based cohort study evaluated the occurrence of pleural empyema in individuals with and without RA. Results showed that patients with RA had a significantly higher risk of developing pleural empyema than those without RA. In accordance with general concepts, the risk of pleural empyema was greater in older persons, men, and individuals with high CCI score and comorbidity of diabetes, asthma/COPD, cancer, and CKD. The risk of pleural empyema was significantly higher in the RA group than in the comparison group after stratification by age, gender, and comorbidity presence. Moreover, ICU admission and 30-day mortality rates for pleural empyema in patients with RA were higher, but not significantly, than in those without RA.

Several studies have mentioned the development of pleural empyema in patients with RA. Walker and Wright [23] have early reported one case of pleural empyema among 19 patients with rheumatoid pleural effusion more than 50 years ago. Then, Jones and Blodgett [16] observed 10 patients with rheumatoid pleural effusion during a 5-year period and found five patients developing pleural empyema. They suggested that pleural empyema frequently occurs in patients with RA. However, Blau [24] failed to find any pleural empyema in 75 thoracentesis procedures carried out in RA patients. He considered that pleural empyema is a far rare occurrence in patients with RA. Recently, an increasing number of empyema cases in RA have been identified; therefore, the association between RA and pleural empyema has been recognized [3,4]. In this study, the incidence of pleural empyema was 23.6/10,000 in RA, which was similar to certain immunocompromised conditions with preexisting pleural effusion (18.2/10,000 in cirrhosis and 23.7/10,000 in end-stage renal disease) [25,26].

It is reasonable that patients with RA who develop pleural empyema may have a poorer prognosis. A previous study [27] showed that polymicrobial infections of pneumonia were more frequent in patients with RA than in those without RA. The mortality rate of pneumonia was significantly higher in patients with RA than in those without RA (11.3% vs 5.0%, *p* < 0.05). The multivariate analysis also showed that RA is an independent risk factor for mortality. However, in this study, the 30-day mortality for pleural empyema was higher in the RA group, although not significant, than in the comparison group. We consider that limited event numbers may have limited the significance of our results.

### 4.1 Strengths

The strength of our study included its use of population-based data to enroll sufficient RA cases (*n* = 29,061) and non-RA controls (*n* = 1,16,244) to evaluate the development of pleural empyema. Taiwan’s NHIRD is one of the largest nationwide population databases in the world, and no difference was found in the demographic distribution between LHID2000 and the original NHIRD. In addition, the universal coverage (>99.9%) in the insurance program reduces barriers to healthcare access for all citizens regardless of socioeconomic background and residential location [28]. By using the NHIRD, we were able to reflect a “real-world” scenario in which RA, pleural empyema, and all other comorbidities were directly diagnosed during medical consultation.

### 4.2 Limitations

Certain limitations to our findings should be considered. First, RA, pleural empyema, and comorbidities were diagnosed using the ICD format, which relies on the performance of specialist physicians. Audits are regularly performed to prevent misdiagnoses and negligence. Second, the NHI research center did not survey comprehensive data, such as occupation, smoking habits, alcohol consumption, diet style, physical activity, body mass index, environmental exposure, and family history, which are possible confounding factors. Third, the disease status and treatment status of the RA patients were unknown. This prevented us from further analyzing the impact of RA severity and medications on the risk of pleural empyema. Fourth, several relevant clinical variables such as physical findings, laboratory data, culture results, and image reports were unavailable in the database. Finally, despite a meticulous study design with an adequate control of confounding factors, a key limitation of this study is the potential for bias because of possible unmeasured or unknown confounders.

## 5 Conclusion

Patients with RA are at a greater risk of developing pleural empyema than those without RA. The risk of pleural empyema remained high when stratified by age, gender, and comorbidity presence. The ICU admission and 30-day mortality rates for pleural empyema in patients with RA were higher, but not significantly, than in those without RA. Additional evidence is needed to support this association, and further investigations are required to clarify the detailed mechanisms involved.
